# Oxidative Stability and Quality Deterioration of *Ganoderma lucidum* Spore Oil Under Accelerated Storage Conditions

**DOI:** 10.3390/foods15162776

**Published:** 2026-08-07

**Authors:** Zhihao Ge, Xuebing Zhang, Lingyan Zhang, Xingfeng Guo, Xiuzhu Yu, Jia Chen

**Affiliations:** 1 Shandong Key Laboratory of Applied Technology for Protein and Peptide Drugs, School of Pharmaceutical Sciences and Food Engineering, Liaocheng University, 1 Hunan Road, Liaocheng 252000, China; 2 Engineering Research Center of Grain and Oil Functionalized Processing in Universities of Shaanxi Province, College of Food Science and Engineering, Northwest A&F University, 22 Xinong Road, Yangling 712100, China

**Keywords:** *Ganoderma lucidum* spore oil, oxidative stability, glycerol core aldehydes, phytosterols

## Abstract

Prior studies on *Ganoderma lucidum* spore oil (GLSO) rarely systematically explored its storage oxidative deterioration mechanism via multi-marker combined characterization. This study innovatively integrated the Schaal oven accelerated storage test and dual-NMR detection (LF-NMR and ^1^H NMR) to evaluate the oxidative stability of GLSO over 60 d. Dynamic changes in routine oxidation indices, glycerol core aldehydes (GCAs), phytosterols and their triterpenoid biosynthetic precursors, fatty acids and volatile compounds were synchronously tracked. Results revealed complex fluctuating accumulation of primary and secondary oxidation products; non-volatile GCAs exhibited continuous monotonic growth and acted as reliable markers for severe GLSO oxidation. Unsaturated fatty acids and phytosterols and their triterpenoid biosynthetic precursors degraded in a structure-dependent pattern, with phytosterols and their triterpenoid biosynthetic precursors degradation sensitivity ranked as squalene > lanosterol > β-sitosterol. Correlation analysis clarified the intrinsic linkage between fatty acid substrates and oxidation derivatives. This work innovatively reveals the complete lipid oxidation pathway of GLSO, offering novel theoretical references and multi-index evaluation strategies for quality control and preservation of high-grade GLSO products.

## 1. Introduction

*Ganoderma lucidum*, a traditional Chinese medicinal herb with a 2000-year history of medicinal use in China and throughout Asia, is also known as lingzhi. Traditional Chinese medicine regards *Ganoderma lucidum* as a panacea with the ability to nourish the body and promote longevity [1,2]. *Ganoderma lucidum* spore powder is a concentrated powder made from the mature reproductive cells released during the mushroom’s maturation stage. It contains all of the mushroom’s genetic active substances and requires cell wall disruption to facilitate the release of nutrients [3].

*Ganoderma lucidum* spore oil (GLSO) is a fat-soluble extract obtained from cell-wall-broken *Ganoderma* spores using supercritical carbon dioxide extraction technology, this method offers high extraction efficiency and better preserves active components [3,4]. Triglycerides account for more than 70% of all fat-soluble components in GLSO. Monounsaturated fatty acids account for 66.5% to 71.7% of the total fatty acids, making them the primary component, with oleic acid being the predominant one. Saturated fatty acids account for 22.0% to 30.4%, with palmitic acid and stearic acid being the predominant ones [5,6]. In addition, it contains triterpenes (5–30%), steroids, and other characteristic components of *Ganoderma lucidum*. GLSO soft capsules have been included in the Special Food Catalog by the State Administration for Market Regulation of China. Pharmacological studies have shown that GLSO exerts antitumor effects by promoting tumor cell apoptosis, regulating the cell cycle, and interacting with chemotherapeutic agents [7,8,9,10], antioxidant [11], lipid metabolism regulation [12], and neuroprotection [3,13]. This subject has become a prominent research area in natural medicines and health supplements, possessing significant medicinal value and broad development prospects. However, recent research on GLSO has largely focused on its composition and pharmacological effects, while neglecting the oxidative reactions of GLSO as a lipid [6]. Notably, lipid oxidation drastically damages the sensory quality, flavor and shelf life of edible oils. Accordingly, exploring the oxidative characteristics of GLSO is vital to guarantee its quality and safety, and oxidative stability acts as a core metric for oil quality evaluation and shelf-life prediction.

Common accelerated oxidation models used to evaluate the oxidative stability of fats and oils primarily include the Rancimat method and the Schaal oven accelerated storage test. Among these, the Rancimat method can only provide a single oxidation endpoint and cannot perform continuous sampling at multiple time points over the long term. The Schaal oven test conducts accelerated oxidation at 63 °C under dark and constant temperature conditions to simulate radical mediated chain reaction pathways that take place during the autoxidation of fats and oils stored at room temperature. The types of oxidation products and the patterns of deterioration closely match those observed during long-term storage at room temperature [14]. Due to its simplicity and reliability, it remains one of the preferred methods for assessing oxidative stability in the fats, oils, and food industries, and is especially well suited for applications requiring rapid comparison of multiple samples or the screening of antioxidants. For instance, a study used the Schaal oven test to determine the oxidation induction period of crude oils from eight oat varieties [15]. The results showed that oat oil exhibits excellent antioxidant stability, successfully distinguished the oxidation trends among different varieties, and effectively reflected changes in oil stability under actual storage conditions.

The objective of this study is to evaluate the oxidative stability of GLSO over 60 d using the Schaal oven test, to elucidate the entire process of lipid oxidative degradation during the storage of GLSO, and to screen for stable biomarkers that characterize deep internal oxidation. Through a 60 d continuous storage experiment, changes in its physicochemical parameters, characteristic oxidation products, and the composition of phytosterols and their triterpenoid biosynthetic precursors and fatty acids were analyzed to elucidate the oxidation mechanism of GLSO under 60 d accelerated storage conditions. To track changes in oil quality during storage, this study measured multiple parameters, including routine chemical indicators, non-volatile secondary oxidation products, GCAs, and volatile oxidation products. Finally, the study explored the correlation between major active phytosterols and their triterpenoid biosynthetic precursors components and lipid oxidation products. Studying the oxidative degradation behavior of GLSO helps reveal the underlying causes of its quality deterioration, provides a basis for the development of preservation technologies, and contributes to the production of high-quality, safe spore oil products with excellent sensory properties.

## 2. Materials and Methods

### 2.1. Materials

GLSO was obtained from Shandong Sanxiu Biotechnology Co., Ltd. (Liaocheng, China), and was extracted by supercritical carbon dioxide extraction. No exogenous antioxidants were added. For chemical analyses, n-alkanes (C7-C30) of chromatographic grade were sourced from Sigma-Aldrich (Shanghai, China). Standard compounds, including gallic acid, α-, β-, γ-, and δ-tocopherols, and β-sitosterol, were obtained from YuanYe Biotechnology Co., Ltd. (Shanghai, China). Methyl pentadecanoate and 2-octanol standards (purity ≥ 98 %) were purchased from Sigma–Aldrich (Shanghai, China). Deuterated chloroform (CDCl3, 99.8 %), LLL (purity ≥ 90 %), and OOO (purity ≥ 99 %) were acquired from Shanghai Energy Chemical Reagent Co., Ltd. (Shanghai, China).

### 2.2. Sample Preparation

GLSO was treated using the Schaal oven test. As a measure of the formation of peroxides during the early stages of oxidation, peroxide value (POV) was determined with reference to American Oil Chemists’ Society (AOCS) Official Method Cd 8b-90 for evaluating the oxidative extent of edible oils. GLSO was placed in a 63 °C electric hot-air oven (DHG-9053A, Yiheng, Shanghai, China) to undergo the Schaal accelerated oxidation test, 20 mL samples were taken every 2 d for 60 d. All oil samples were immediately aliquoted and stored in the dark at −20 °C, pending their transfer to glass beakers for testing of various parameters.

### 2.3. Determination of Chemical Parameters

POV, acid value (AV), *p*-anisidine value (*p*-AnV), conjugated dienes (K232), and conjugated trienes (K268) were carried out according to the AOCS official methods Cd 8b-90, Cd 3d-63, Cd 18–90, Cd 20–91, and Ch 5–91. Details of the test procedures were available in Li et al. [16].

### 2.4. Determination of GCAs

The analysis of GCAs was performed according to the protocol reported by Xu et al. [17]. The 300 mg oil sample was mixed the with internal standard, 2 mL sodium methoxide (Sinopharm, Shanghai, China), and tert-butyl methyl ether (3 mL) in a 15 mL centrifuge tube, neutralized with 100 μL of 0.5 M sulfuric acid–methanol solution, diluted to 3 mL with deionized water, vortexed for 1 min, and centrifuged at 3580× *g* for 10 min. The upper organic layer was collected, dried under nitrogen, re-dissolved in hexane, and filtered through a 0.22 μm membrane filter before analysis.

GCAs were determined using a Thermo Trace 1310-ISQ LT gas chromatography–mass spectrometer (GC-MS) system with a VF-WAXms capillary column (60 m × 0.25 mm, 0.25 μm) and FID (Shimadzu, Kyoto, Japan). The oven temperature program was held at 90 °C for 2 min, ramped to 230 °C at 6 °C/min, then held at 230 °C for 30 min, with helium as carrier gas (1 mL/min). Acquisition was performed in SIM mode with 70 eV EI ionization, transfer line and ion source temperatures were 240 °C and 230 °C, respectively. The content of GCAs was calculated according to Equation (1):

(1) $$m_{i}=\frac{A_{i}}{A_{is}}\times \int_{i}^{\prime }\times \alpha ,$$

where m_i_ is the amount of GCAs (mg); A_i_ represents the area of methyl ester aldehydic acid; A_is_ represents the area of the internal standard; m_is_ is the mass (0.1 mg) of the internal standard in sample; f^’^_i_ is the relative correction factor of 1; and α is methyl ester aldehydic acids to GCAs, α(8-oxo) = 4.41, α(9-oxo) = 4.16.

### 2.5. Determination of Phytosterols and Their Triterpenoid Biosynthetic Precursors

The phytosterols and their triterpenoid biosynthetic precursors’ profile was quantified by GC-MS based on the method reported by Zhang et al. [18]. Analyses were carried out on a Shimadzu GC-2010 GC coupled to a Shimadzu MS-QP 2010 Plus mass selective detector (Shimadzu, Kyoto, Japan). A total of 0.3 g of oil sample was saponified using 10 mL of 2 mol/L ethanolic potassium hydroxide (KOH) solution at 85 °C for 1 h. After saponification, the mixture was extracted with 5 mL of distilled water and 5 mL of n-hexane to isolate the unsaponifiable fraction containing phytosterols and their triterpenoid biosynthetic precursors. The unsaponified fraction was extracted with dichloromethane and incubated in 100 µL MTBSTFA: 1-MIN (95:5, *v*/*v*) at 75 °C for 20 min. Nonsaponified compounds including squalene were retained in the aqueous phase and partitioned into the n-hexane organic layer. A 1 μL aliquot of the organic extract was injected into an SH-Rxi-5Sil MS capillary column (30 m × 0.25 mm × 0.25 μm, Shimadzu, Kyoto, Japan). The temperature settings of the oven are listed below: the temperature was first held at 180 °C for 1 min, then ramped linearly to 300 °C at a rate of 4 °C/min, with a subsequent 5 min hold at 300 °C. Helium was used as the carrier gas at a constant flow rate of 0.7 mL/min, with 5α-cholestanol selected as the internal standard for quantitative calibration.

### 2.6. Determination of Fatty Acid Composition

Fatty acid profiles were analyzed via GC according to the method reported by Chen et al. [19]. Before GC analysis, fatty acids need to be converted to their methyl esters (FAMEs). The specific operation is as follows: take 60 mg of oil sample and mix with 2 mL of internal standard solution (methanol solution containing 5 mg/mL glycerol tridecanoic acid methyl ester), and add 4 mL of isooctane. Subsequently, 200 μL potassium hydroxide methanol solution (2 mol/L) was added for methylation reaction. The mixture was vortexed vigorously for 1 min and left to stand in the dark for 2 h at room temperature. Then 1 g of sodium bisulfite was added and shaken vigorously to neutralize the residual potassium hydroxide. After completing the above steps, transfer the upper liquid to the sample bottle. Analysis was carried out using an 6890 N GC system (Agilent, Santa Clara, CA, USA) equipped with a flame ionization detector and an HP-INNOWAX capillary column (30 m × 0.25 mm, 0.25 μm, Agilent, Santa Clara, CA, USA). The oven temperature program was initiated at 60 °C, ramped to 200 °C at 10 °C/min, then further increased to 240 °C at 8 °C/min, and held constant for 15 min. Retention times of fatty acid methyl esters were compared with those of certified standards to realize compound identification.

### 2.7. Determination of Relaxation Characteristics (T_2_) by LF-NMR

The detection method for LF-NMR is based on Xie et al. [20]. Magnetic resonance imaging was performed using the frequency coil of an LF-NMR analyzer (Niumag, Suzhou, China) at 32 °C. Proton density images of the TPS were obtained with the multiple spin echo (MSE) sequence of the MRI software (Ver.2.3, Niumag, Suzhou, China), under the following scan parameters: field of view = 70 mm × 70 mm, number of averages = 16, read size = 256, phase size = 192, echo time (TE) = 10 ms, and repetition time (TR) = 800 ms.

### 2.8. Determination of Oxidation Products by ^1^H NMR

^1^H NMR measurements were performed on a Bruker Avance 400 MHz instrument (Bruker, Billerica, MA, USA) to analyze oxidation products, as per the procedure documented by Wu et al. [21]. Each sample (50 μL) was diluted in 400 μL CDCl_3_ prior to testing. The levels of oxidation products in GLSO were calculated using the following Equation (2) and expressed in the unit of mmol/mol

(2) $$\left[OP(mmol/mol\;TAG)]=1000\times [(A_{\mathrm{OP}}/n)/(A_{I}/4)\right]$$

where A_OP_ is the oxidation product signal, n is the number of protons from the signal, and A_I_ is the area of protons at sn-1 and sn-3 positions in TAGs’ glycerol backbone.

### 2.9. Determination of Volatile Component

The analysis of volatile compounds in GLSO was performed using headspace solid-phase microextraction (HS-SPME) combined with GC-MS, following the protocol of Zhang et al. [22]. A total of 4 g of sample was transferred to a 20 mL headspace vial, spiked with 4 μL of 2-octanol (0.49 mg/mL internal standard), and equilibrated at 50 °C for 30 min. The SPME fiber was desorbed in the GC injector at 250 °C for 3 min under splitless mode, with helium as the carrier gas at 1.0 mL/min. The oven temperature program was held at 40 °C for 3 min, ramped to 120 °C at 4 °C/min, then to 240 °C at 6 °C/min maintained for 9 min. Volatile compounds were identified by matching mass spectra and retention indices with the NIST 14 library (85–100%). All analyses were performed in triplicate, and results are presented as mean values.

### 2.10. Statistical Analysis

All determinations were conducted three times, independently. All results are shown as mean ± standard deviation (SD). One-way analysis of variance (ANOVA) together with Duncan’s multiple-range test (*p* < 0.05) was adopted to examine statistical differences between groups, with SPSS 19.0 (IBM, Armonk, NY, USA) as the analysis software.

## 3. Results and Discussion

### 3.1. Changes in POV, K232, K268, p-AnV and AV

Lipid oxidation is a complex free-radical chain reaction process that significantly affects the sensory quality, nutritional value, and even the safety of food [23]. POV, K232, K268, *p*-AnV, and AV are key indicators for assessing the degree of oxidation and quality deterioration in edible oils and fats; they characterize the products of different stages in the lipid oxidation and hydrolysis processes, respectively, and together form a comprehensive system for monitoring quality deterioration.

**Figure 1:**
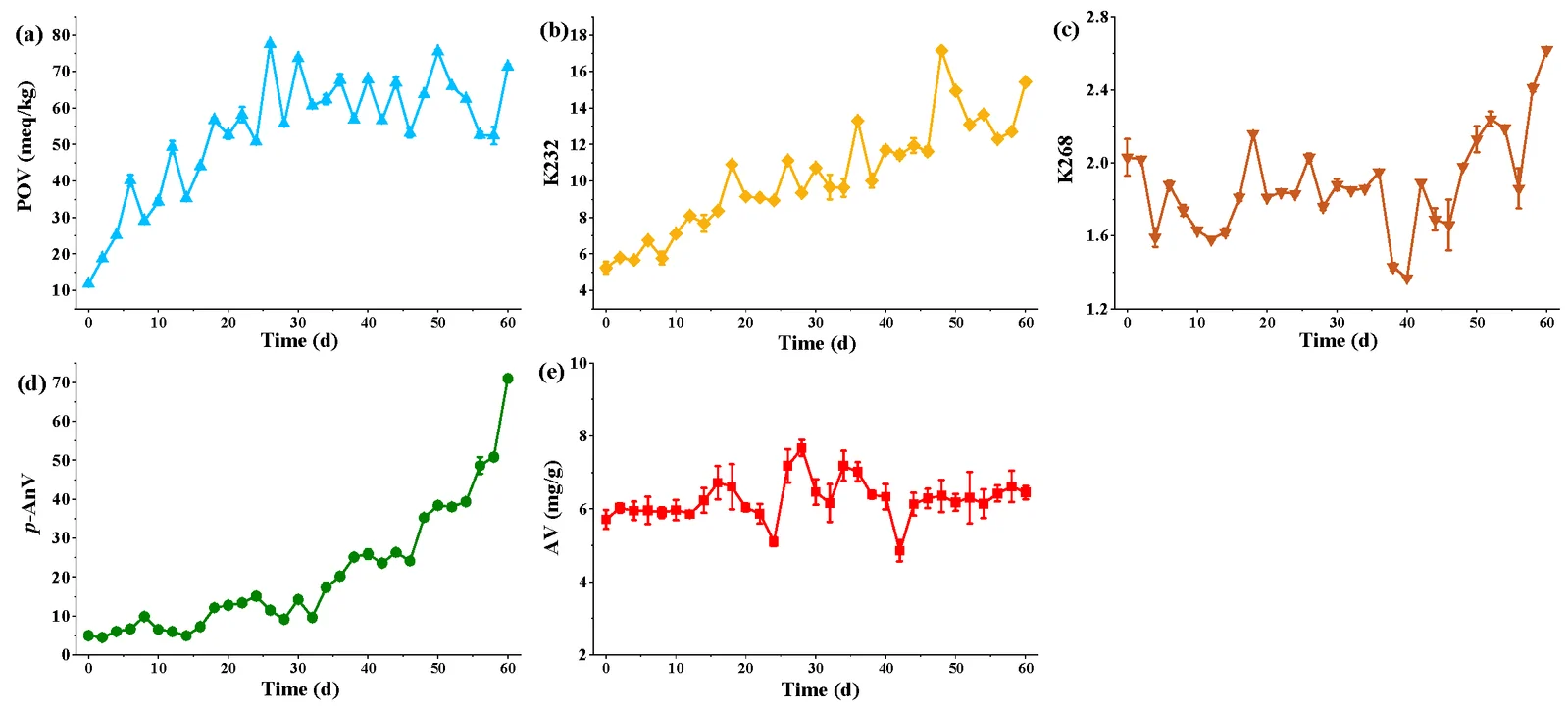
Evolution of (**a**) POV, (**b**) K232, (**c**) K268, (**d**) *p*-AnV, and (**e**) AV in GLSO during 60 d storage period.

Dynamic changes in lipid oxidation indicators during storage are depicted in Figure 1. The abundance of hydroperoxides in GLSO was determined by POV, which represents a reliable measure of primary lipid oxidation [24]. Over 60 d of storage, the POV showed pronounced fluctuations with multiple distinct peaks, rising rapidly from 11.82 meq/kg at 0 d to a maximum of 77.6 meq/kg at 26 d. This is because hydrogen peroxides are extremely unstable at high temperatures. On the one hand, they are formed by the oxidation of unsaturated fatty acids, and on the other hand, they rapidly decompose into aldehydes, ketones, alcohols, and other compounds. When the formation rate exceeds the decomposition rate, the POV rises; when the decomposition rate exceeds the formation rate, the POV falls [25,26]. K232 and K268 are two key ultraviolet absorption spectral parameters used to assess the oxidative stability and degree of quality deterioration in edible oils. The changes in K232 and K268 are shown in Figure 1b,c. The concentration of K232 exhibits a nonlinear, fluctuating increase with prolonged storage time. This is because conjugated dienes react with oxygen to form more unstable lipid hydroperoxides (LOOH) [27]. These hydroperoxides decompose very easily, generating large amounts of alkoxy radicals (LO·) and hydroxyl radicals (·OH), thereby triggering new chain reactions and breaking down into a series of secondary oxidation products [28]. As K232 are continuously consumed to generate these downstream products, their concentration begins to decline after reaching a peak. The primary reason for the fluctuations in K268 levels is that conjugated trienes are highly unstable intermediates in the initial oxidation reaction.

The *p*-AnV serves as a primary measure of oil stability by quantifying aldehydes, which are the main byproducts of lipid oxidation. It displayed an overall upward trend with minor fluctuations; it remained at a low baseline initially, and then accelerated sharply, reaching the peak at 60 d, which reflected the progressive accumulation of secondary oxidation products derived from hydroperoxide breakdown. Together, these trends highlight the complex interplay between primary and secondary lipid oxidation processes; primary oxidation products are highly unstable due to the weak bond energy of the peroxide bond (-O-O-) in their molecular structure, leading to the formation of highly reactive radical intermediates. These intermediates are then further converted into various secondary oxidation products through reactions such as β-cleavage, cyclization, or rearrangement [29]. The AV is a classic indicator used to assess the degree of hydrolytic rancidity in fats and oils; it reflects the level of free fatty acids produced by the hydrolysis of triglycerides. It fluctuated throughout the period, initially increasing from 5.71 mg/g at 0 d to a peak of 7.67 mg/g at 28 d, followed by sharp declines at 32 d and 42 d (to 6.16 and 4.85 mg/g, respectively), before stabilizing between 6.0 and 7.0 mg/g in the final stages. Specifically, AV generally shows an upward trend during the early stages of oxidation, driven primarily by the hydrolysis of triglycerides. However, AV does not rise in a linear fashion in actual systems; one of the direct causes of its decline is the further degradation of free fatty acids which serve as oxidation substrates into secondary products such as aldehydes, ketones, and epoxides. Consequently, variations in AV follow a fluctuating pattern [30].

### 3.2. Changes in GCAs

**Figure 2:**
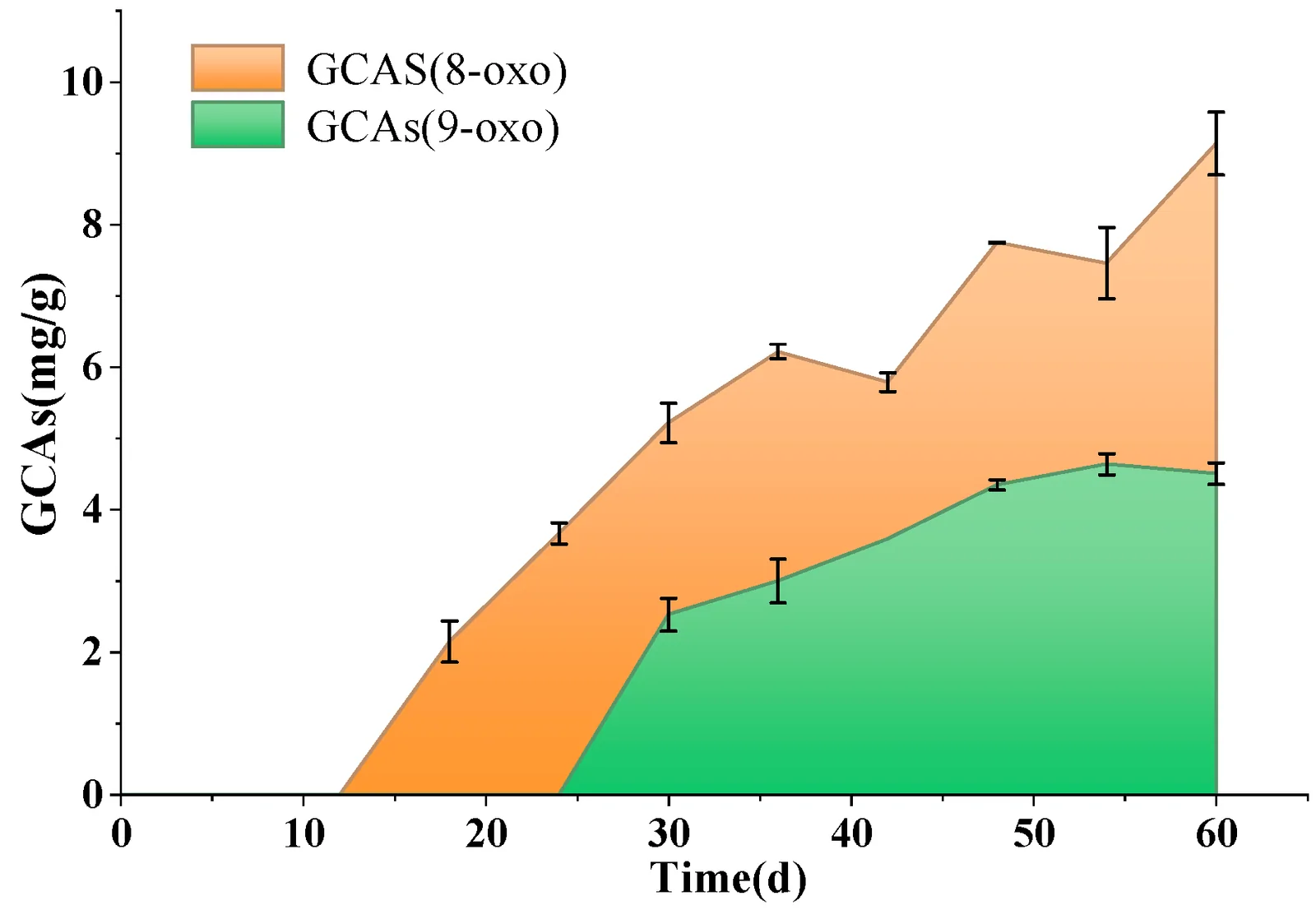
Changes in GCAs of GLSO during 60 d storage period.

GCAs, as non-volatile toxic secondary products generated by lipid oxidation, are characterized structurally by the covalent binding of aldehydes to the glycerol backbone through acyl groups, forming macromolecular bound oxidation products. These compounds are highly fat-soluble and do not undergo volatilization loss, they can objectively reflect the actual degree of oxidative accumulation within fats and oils and can therefore be regarded as markers of oxidation [31]. This study primarily examines changes in GCAs (8-oxo) and GCAs (9-oxo). Among them, GCAs (9-oxo), which can originate from the β-scission of hydroperoxides of oleic acid, linoleic acid, and linolenic acid, accounts for 60% of total GCAs and is the most predominant component, while GCAs (8-oxo), which can only be generated from oleic acid, are present in relatively lower amounts [17,32]. As shown in Figure 2, the contents of GCAs (8-oxo) and GCAs (9-oxo) exhibited a significant time-dependent increase during storage. Experimental data reveal that the accumulation rate of GCAs (8-oxo) displays distinct phase characteristics, potentially corresponding to the oxidation induction period, during which primary oxidation products have not undergone large-scale decomposition. Starting from 18 d, the generation rate of GCAs (8-oxo) significantly accelerates, reaching a peak on 60 d and continuing to increase, whereas GCAs (9-oxo) stabilize at a relatively stable level. This exponential growth pattern aligns with the findings indicating that, as secondary oxidation products, GCAs primarily form due to the β-scission of hydroperoxide alkoxy radicals from UFAs. Notably, the final content of GCAs (8-oxo) is significantly higher than that of GCAs (9-oxo), the main reason is that GCAs (8-oxo) are derived from oleic acid, which accounts for over 60% of the total fatty acids in GLSO. In contrast, GCAs (9-oxo) originate from linoleic acid and linolenic acid [17].

Unlike small aldehydes that typically decrease due to volatilization during the later stages of oxidation, GCAs in this study exhibited a continuous and monotonous increase throughout the entire storage period, without showing a downward trend. This phenomenon can be attributed to the unique structural characteristics of GCAs. According to the mechanistic analysis by Xu et al. [17,30], GCAs are non-volatile compounds, with their aldehyde groups firmly attached to the glycerol backbone via ester bonds. Their high molecular weight characteristics make it difficult for them to escape from the oil and fat system. Therefore, GCAs can serve as a stable oxidative marker, accurately reflecting the degree of internal oxidative deterioration in oils and fats. Furthermore, high concentration levels (0.81 mg/g) not only indicate a deep oxidative state but also suggest potential polymerization risks. As pointed out by Chen et al. [30], these accumulated GCAs possess high reactivity and may act as intermediates, further reacting with other oxidative products through their aldehyde groups to form dimers or high molecular weight polymers. This also explains why the accumulation of GCAs during the later stages of oxidation is often accompanied by an increase in oil and fat viscosity and the total amount of polar compounds.

### 3.3. Changes in Phytosterols and Their Triterpenoid Biosynthetic Precursors

**Figure 3:**
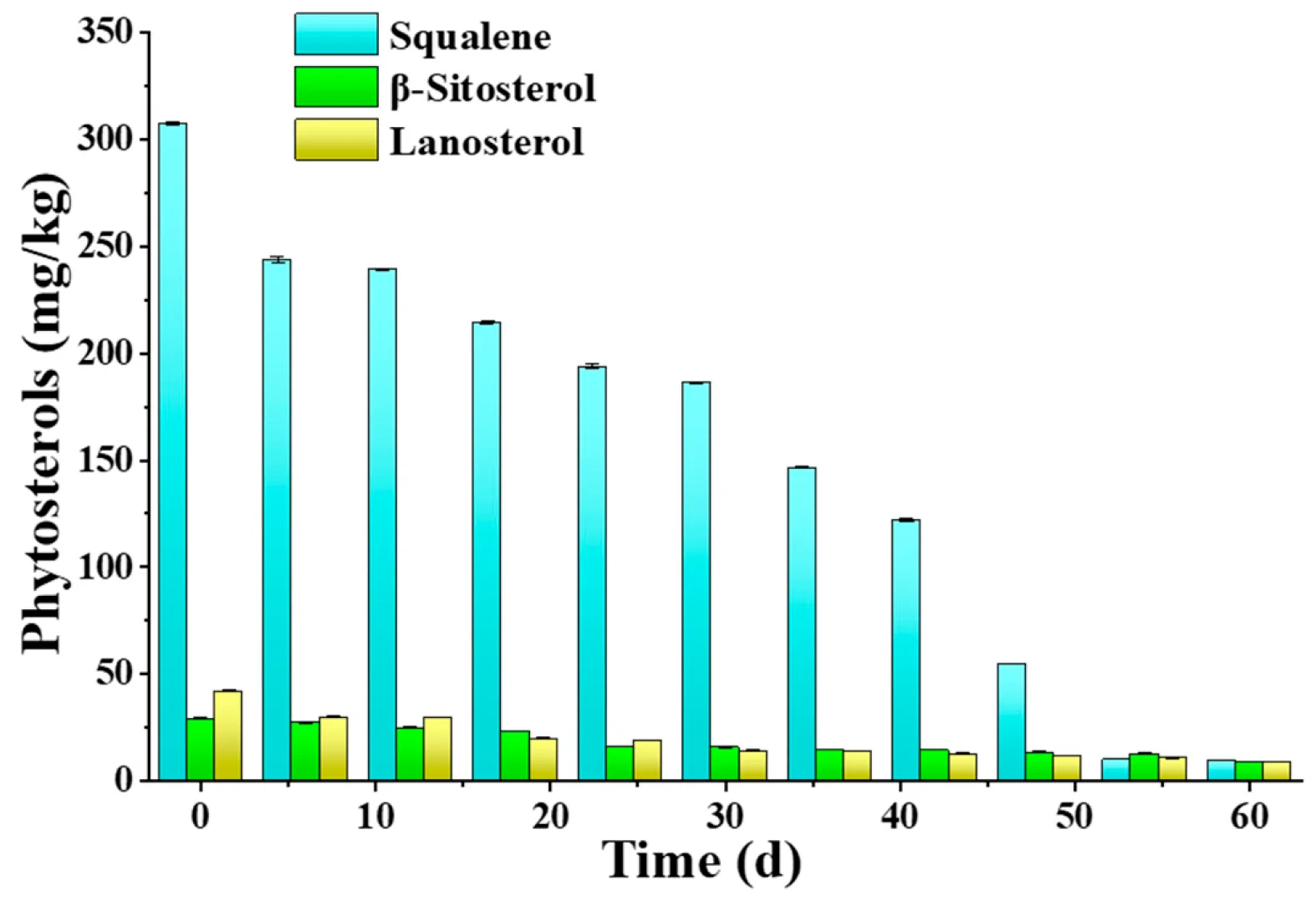
The changes in contents of phytosterols and their triterpenoid biosynthetic precursors in GLSO during 60 d storage period.

The unsaponifiable fraction of GLSO is rich in oxidation-prone triterpenoid components, including typical phytosterols and their biosynthetic precursors. Among them, β-sitosterol belongs to classic phytosterols, squalene is an open-chain triterpene hydrocarbon as a key upstream precursor for sterol synthesis, and lanosterol is a tetracyclic triterpenoid intermediate in the phytosterol biosynthesis pathway [33]. Figure 3 illustrates the dynamic content changes in these triterpenoid components during the 60 d storage period. The results suggest that all components exhibited significant time-dependent degradation. Squalene, with the highest initial content (307.30 mg/kg), depleted rapidly to 9.83 mg/kg by 60 d. Conversely, β-sitosterol (29.62 to 9.29 mg/kg) was the most stable, showing a gradual decline with the sharpest decrease between 24 d and 30 d. Lanosterol (initially 42.49 mg/kg) displayed a distinct two-stage profile, falling sharply to 20.27 mg/kg within 18 d, and subsequently hovering between 8.99 and 19.22 mg/kg until 60 d. Overall, the degradation sensitivity of the three components was in the order of squalene > lanosterol > β-sitosterol, which is closely related to their molecular structures. Squalene, as an open-chain triterpene hydrocarbon containing six double bonds, is prone to auto-oxidation. Lanosterol exhibits moderate stability due to the unsaturated bonds in the steroid core, while β-sitosterol demonstrates the strongest antioxidant degradation ability due to its fully saturated side chains and stable steroid core. Overall, the thermal stability of the phytosterols and their triterpenoid biosynthetic precursors is ranked as β-sitosterol > lanosterol > squalene, primarily due to differences in the number, position, and stereochemistry of the double bonds in their molecular skeletons. β-sitosterol exhibits relatively high structural stability because its side chains are saturated and its core contains only one double bond. Squalene, a linear triterpene containing six non-conjugated double bonds, is chemically highly reactive and is extremely prone to auto-oxidation. In contrast, lanosterol, a tetracyclic sterol precursor, exhibits intermediate stability between the two sterols [34,35].

### 3.4. Changes in Fatty Acid Composition

Table 1 shows the four major fatty acids in GLSO, oleic acid (C18:1), a monounsaturated fatty acid, was identified as the predominant component, representing 65% of the lipid profile. This was followed by the polyunsaturated linoleic acid (C18:2) at 13.6%. The saturated fatty acids (SFAs), primarily comprising palmitic acid (C16:0) and stearic acid (C18:0), constituted roughly 20% of the total content. The ratios of the four fatty acids in the GLSO analyzed in this study were similar to those determined in previous analyses [5,6]. Table 1 illustrates the fatty acid constituent profiles of GLSO. During the storage period of 0 to 60 d, the content changes in two saturated fatty acids and two UFAs in oils and fats exhibited significant structure-dependent differences. This is because different types of fatty acids have varying levels of antioxidant capacity [36]. Because the double bonds in polyunsaturated fatty acids may form epoxides and furan compounds under free radical attack, these cyclization products may be converted into saturated cycloalkanes or branched-chain saturated fatty acids in subsequent reactions, leading to an increase in saturated fatty acids [37]. Palmitic acid continuously rose to a stable peak of 16.06 g/100 g (42 d). Stearic acid peaked earlier at 4.62 g/100 g (30 d) before declining, likely due to its long chains participating in polymerization and esterification networks [38]. Conversely, UFAs degraded via free-radical auto-oxidation, correlating with their degree of unsaturation. Linoleic acid dropped to 11.31 g/100 g at 48 d, yielding aldehydes and ketones responsible for rancidity [39]. Oleic acid showed moderate stability with nonlinear degradation mainly between 18 d and 30 d. The apparent rise in oleic acid content from 36 d to 48 d is attributed to differential oxidation rates driven by structural variations among fatty acids. Linoleic acid degrades at a far higher rate than oleic acid. Between 36 d and 48 d, linoleic acid is rapidly broken down into volatile secondary products such as hexanal, which volatilize and escape from the open system, leading to a shrinkage of the total pool of detectable intact fatty acids. Since oleic acid undergoes degradation at a much slower pace, its relative proportion increases passively [40]. Ultimately, polyunsaturated fatty acid (PUFA) oxidation is the root cause of quality deterioration, indirectly evidenced by the relative accumulation of SFAs. The above changes indicate that the oxidation of PUFAs is the main cause of quality deterioration during storage, while the accumulation of saturated fatty acids indirectly reflects the loss of unsaturated components.

### 3.5. Measurement of Relaxation Characteristics (T_2_) by LF-NMR

LF-NMR stands out as a powerful analytical technique in food science, owing to its non-invasive, non-destructive advantages and superior discriminative performance [41]. Table 2 presents the T_2_ relaxation time data for GLSO at different storage durations. The multicomponent relaxation spectrum of the GLSO during the oxidation process consists of two peaks, indicating the presence of two states of hydrogen protons in GLSO. The T_2_ relaxation time reflects changes in nuclear spin types and their physicochemical environment. Weak T_21_ peaks and main T_22_ peaks, which are associated with the composition of SFAs and UFAs, were identified, with their peak area percentages recorded as S_21_ and S_22_, respectively. T_21_ is associated with polar oxidation products, particularly short-chain aldehydes and free fatty acids, and its signal is primarily concentrated in the T_21_ region. T_22_ corresponds to the SFA fraction, and its peak area, S_22_, reflects the relative abundances of different types of fatty acids [42]. Oxidation and decomposition reactions during storage are often accompanied by the formation of substances such as aldehydes, ketones, and alcohols [43]. As these small molecules accumulate to a certain concentration, enhanced intermolecular interactions increase the T_21_ signal intensity. Concurrently, molecules such as aldehydes and ketones can combine with other free molecules in the GLSO to form monoglyceride, diglyceride, and triglyceride derivatives. These substances possess strong polarity and intermolecular interactions, which affect the relaxation process of hydrogen protons in a magnetic field, thereby leading to overall changes in the T_2_ spectrum. Furthermore, as storage time increases, the larger the peak area ratio becomes, suggesting that more hydrogen protons in the sample are generating this type of relaxation signal. The T_21_ peak is likely related to the NMR response of hydrogen protons contained in components of the oil with relatively high molecular weight and strong polarity. As temperature rises and oxidation time increases, the content of large-molecule polar compounds in the oil rapidly increases, manifesting as a continuous increase in S_21_ [38].

### 3.6. Analysis of ^1^H NMR

**Figure 4:**
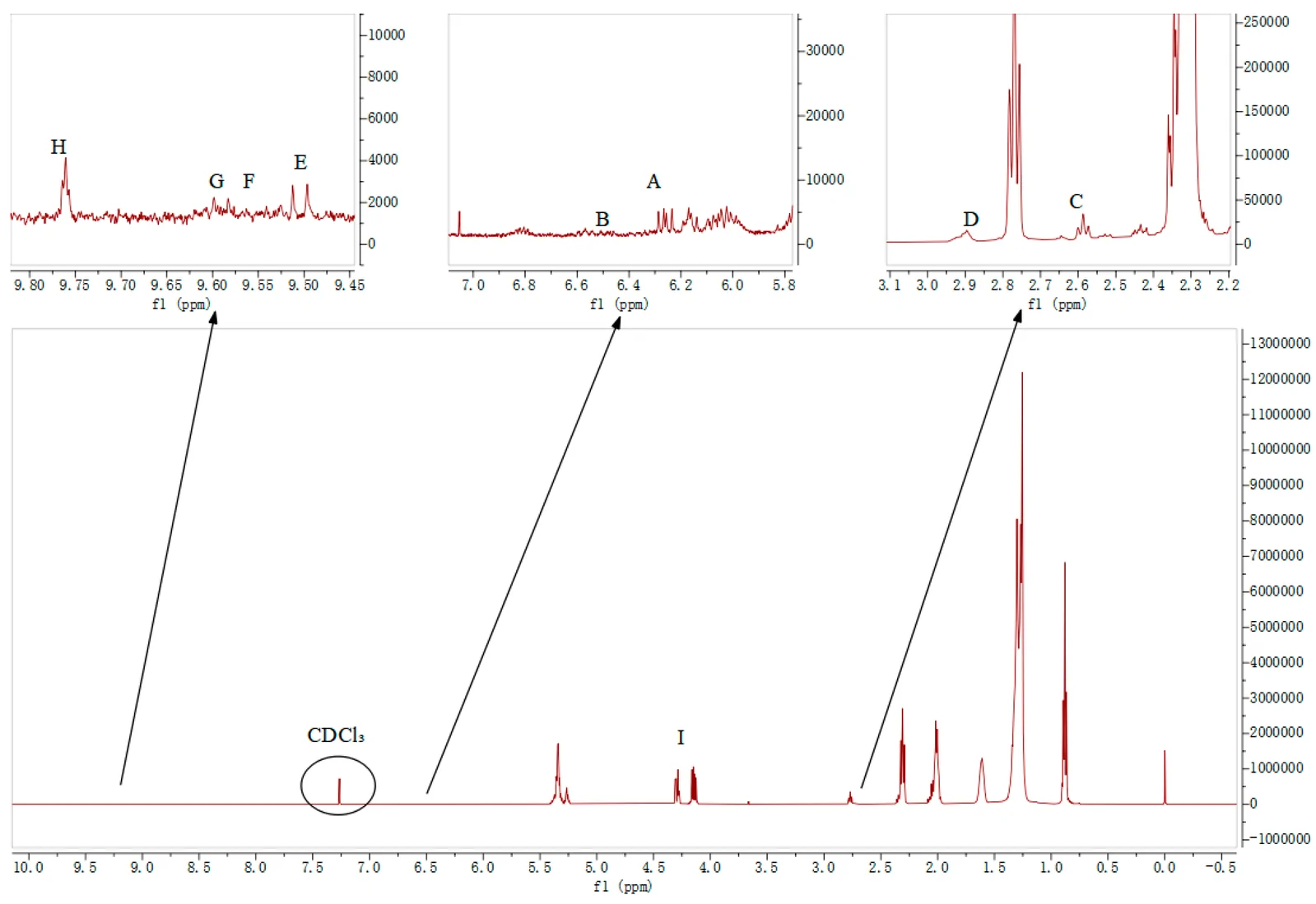
^1^H NMR spectrum of GLSO after 60 d of Schaal oven test together with the enlargement of certain signals.

**Figure 5:**
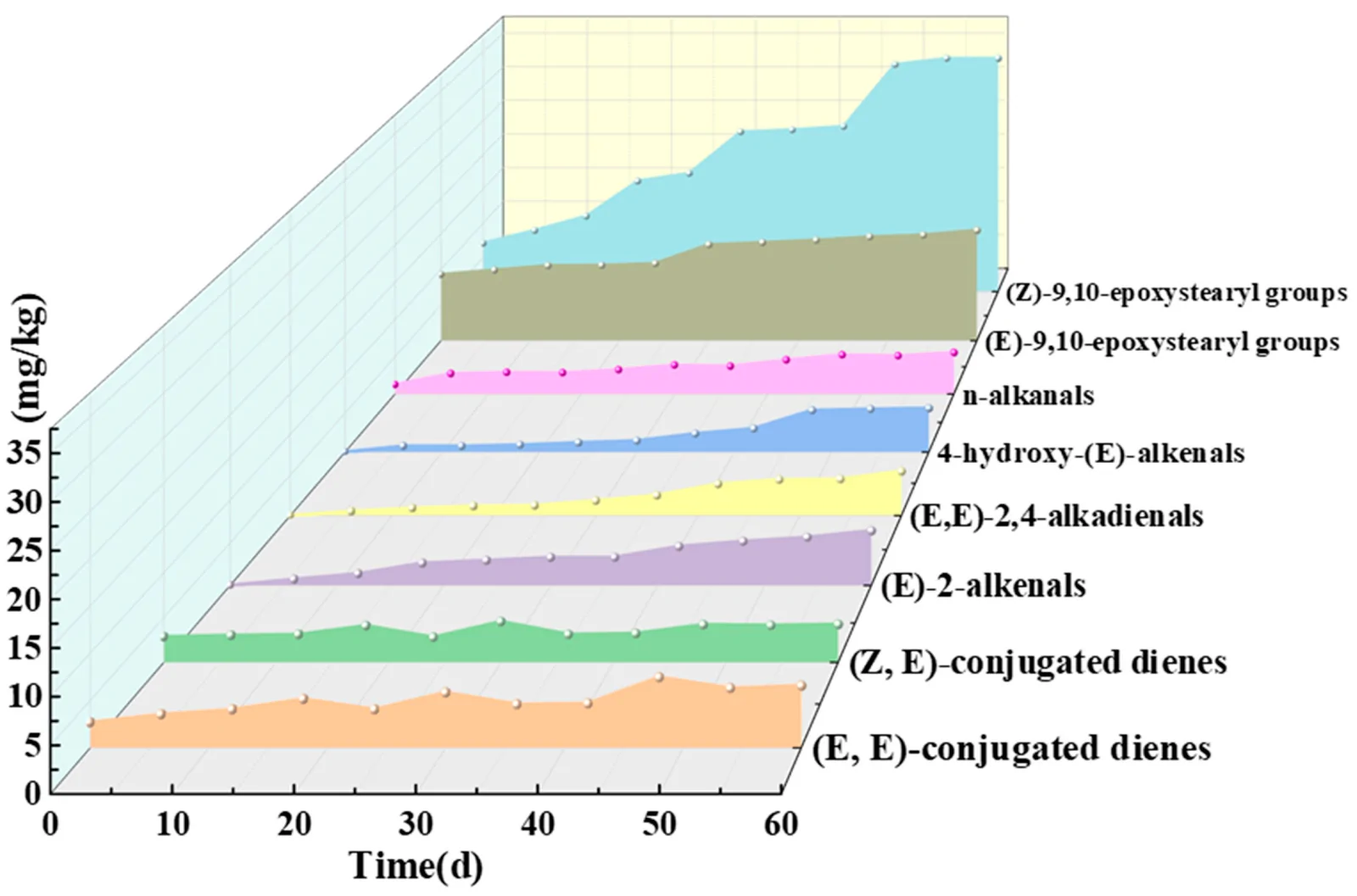
Changes in volatile oxidation products of GLSO during a 60 d storage period.

^1^H NMR spectroscopy is an essential analytical technique for the characterization of food lipids and fats. This technique offers notable advantages including non-destructive detection, low sample dosage, straightforward pretreatment and short analysis time [44]. One of the changes that occurs during the oxidation of GLSO is the formation of peroxides in the acyl structure [45]. The major oxidation products (E,E)-conjugated dienes and (Z,E)-conjugated dienes, as well as the minor oxidation products (E)-9,10-epoxystearyl groups, (Z)-9,10-epoxystearyl groups, (E)-2-alkenals, (E,E)-2,4-alkadienals, 4-hydroxy-(E)-alkenals, and n-alkanals. Proton chemical shifts (δH) of GLSO oxidative and hydrolytic products are given in Figure 4 and Table 3. The chemical shift values of hydrogen atoms in the major oxidation products. (E,E)-conjugated dienes and (Z,E)-conjugated dienes, are 6.27 and 6.49, respectively. The chemical shift values of hydrogen atoms in secondary oxidation products (aldehydes) range from 9.49 to 9.78. In contrast, the (E)-9,10-epoxystearyl groups and (Z)-9,10-epoxystearyl groups were present in only small amounts among the secondary oxidation products, at 2.63% and 2.88%, respectively. As shown in Figure 5, the content of primary oxidation products exhibits a fluctuating upward trend. The content of (E,E)-conjugated dienes reached its peak on 48 d. In contrast, the fluctuating increase in the content of (Z,E)-conjugated dienes is primarily driven by the intermittent generation of free-radical chain reactions during the early stages of oxidation, coupled with dynamic changes in oxygen concentration and antioxidant depletion. The content of secondary oxidation products continues to rise with increasing storage time, with the concentration of (Z)-9,10-epoxystearyl groups being significantly higher than that of other compounds. During lipid auto-oxidation, hydrogen abstraction from polyunsaturated fatty acids produces alkyl radicals that rearrange to form conjugated dienes, leading to continuous accumulation of diene structures and elevated NMR proton signal intensity. Nevertheless, conjugated dienes are highly reactive primary intermediates that readily combine with oxygen to form lipid hydroperoxides measured by POV. These thermally unstable hydroperoxides undergo alkoxy radical β-scission, which consumes conjugated diene precursors and generates abundant secondary oxidation products, causing periodic declines in diene concentration and resulting in their overall fluctuating growth [46].

### 3.7. Analysis of Volatile Compounds in GLSO

During the oxidation of fats and oils, carbonyl compounds play a key role in the formation of their flavor, these compounds typically consist of aldehydes, ketones, and alcohols. Extended storage promotes the autoxidation reaction of GLSO, accompanied by the generation of abundant volatile constituents. These compounds include 13 aldehydes, four ketones, four alcohols, two alkanes, three acids, and one furan (Table 4), these substances can serve as markers of the gradual oxidation of fats and lipids. Distinct differences in volatile components were observed among the types of GLSO. The following aldehyde were detected in the oil samples: pentanal, 2-methyl-pentanal, hexanal, 2-hexenal, Heptanal, (E)-2-heptenal, octanal, (E)-2-octenal, (E)-4-nonenal, nonanal, (E)-2-nonenal, decanal, and (E,Z)-2,4-decadienal. Hexanal is the primary volatile compound. The accumulation patterns of such compounds are typically associated with the extensive degradation of PUFAs through hydroperoxide-mediated cleavage [47]. Although most aldehydes contribute to the aromatic profile of oils, some also impart grassy and nutty characteristics. Therefore, aldehydes play a critical role in shaping the flavor profile of oils [48].

Among the four ketone compounds, the trend of 1-octen-3-one is particularly distinctive: it was virtually undetectable during the first 30 d of storage, peaked at 1.14 mg/kg on 48 d, and then rapidly dropped to 0.04 mg/kg by 60 d. This pulsed fluctuation in concentration is a typical characteristic of periodic bursts in radical chain reactions and is most likely triggered by the replenishment of oxygen in the headspace gas and the depletion of local endogenous antioxidants in the fat [49]. The four alcohols detected were 1-pentanol, 1-heptanol, 1-octen-3-ol, and 1-octanol, with their overall concentrations showing an upward trend. The formation of alcohols stems from the decomposition of hydroperoxides (ROOH), which are products of primary oxidation when ROOH undergoes homolytic cleavage to form an alkoxy radical (RO·); if this radical abstracts a hydrogen atom from a neighboring molecule, it stabilizes to form the corresponding alcohol. Changes in aldehydes, ketones, alcohols, and alkanes provide important scientific evidence for a deeper understanding of the storage stability and quality deterioration mechanisms of GLSO, as well as for the development of corresponding preservation strategies.

### 3.8. Correlation Analysis of the Lipid Oxidation Products

**Figure 6:**
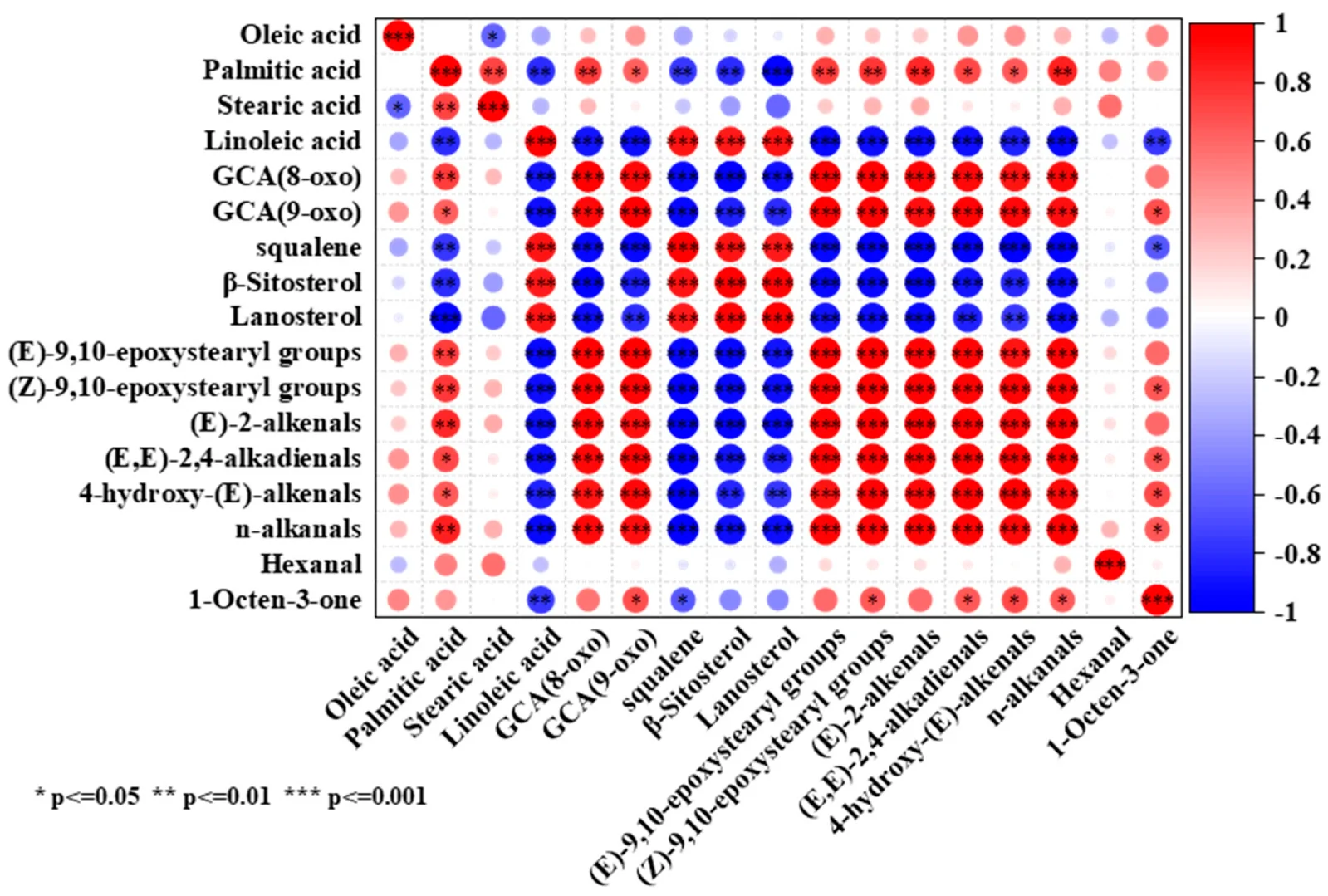
Pearson correlation analysis of the lipid oxidation products.

The study examined the relationships among the oxidation products in GLSO at different storage times. The results are displayed in Figure 6. GCAs (8-oxo) and GCAs (9-oxo) showed a positive correlation with (E)-9,10-epoxystearyl groups, (Z)-9,10-epoxystearyl groups, (E)-2-alkenals, (E,E)-2,4-alkadienals, 4-hydroxy-(E)-alkenals, and n-alkanals (*p* < 0.05). These may be byproducts generated when UFAs undergo common free-radical chain reactions and subsequent hydroperoxide degradation pathways under high temperatures or oxidative stress; their formation rates are primarily controlled by the degree of oxidation of the UFAs, and thus tend to show consistency. In this experiment, linoleic acid showed a positive correlation with squalene, β-sitosterol, and lanosterol, while it exhibited a negative correlation with (E)-9,10-epoxystearyl groups, (Z)-9,10-epoxystearyl groups, (E)-2-alkenals, (E,E)-2,4-alkadienals, 4-hydroxy-(E)-alkenals, and n-alkanals (*p* < 0.05). This is because linoleic acid serves as the primary precursor substrate for these oxidation products; it is continuously consumed during the oxidation process while generating the corresponding secondary oxidation products, resulting in a negative correlation between their trends. Interestingly, Squalene, β-sitosterol, and lanosterol show a negative correlation with (E)-9,10-epoxystearyl groups, (Z)-9,10-epoxystearyl groups, (E)-2-alkenals, (E,E)-2,4-alkadienals, 4-hydroxy-(E)-alkenals, and n-alkanals (*p* < 0.05).

## 4. Conclusions

This study systematically evaluated the oxidative stability and degradation mechanism of GLSO at 60 days using the Schaal oven test method. Lipid auto-oxidation of GLSO can be divided into primary and secondary oxidation stages: the primary stage (0–26 d) is dominated by free-radical chain reactions producing lipid hydroperoxides, K232 and K268, with continuously rising POV and low accumulation of secondary oxidation products. After 26 d, LOOH massive decomposition drives the secondary oxidation dominant stage, generating abundant aldehydes, epoxides, volatile rancid substances and non-volatile GCAs. Specifically, fluctuations and continuous increases in AV, POV, *p*-AnV, alongside K232 and K268, explained the evolutionary patterns of primary oxidation, secondary oxidation, and hydrolysis reactions which occur simultaneously and counterbalance one another during the storage of GLSO. Among secondary products, GCAs accumulated monotonically; due to their polymerization potential, GCAs serve as stable markers for deep internal oxidation. Concurrently, phytosterols and their triterpenoid biosynthetic precursors degradation occurred in a structure-dependent manner. Combining LF-NMR and ^1^H NMR techniques further confirmed that, at the molecular level, the formation of oxidation products and the accumulation of these substances significantly altered the relaxation characteristics of hydrogen protons. Furthermore, the aldehydes formed by the oxidation of linoleic acid such as hexanal and nonanal are among the numerous volatile compounds produced during the oxidation process. These compounds cause changes in the flavor of fats and oils; therefore, they serve as standard biomarkers for evaluating flavor deterioration and the degree of oxidation. In summary, this study has laid a solid theoretical foundation for the preservation strategies and quality control of GLSO.

## Figures and Tables

**Table 1:** Fatty acid composition (g/100 g) of GLSO during 60 d storage period.

Time (d)	Fatty Acid Composition (g/100 g)			
	C16:0	C18:0	C18:1	C18:2
0	15.04 ± 0.01 e	3.92 ± 0.00 e	63.28 ± 0.02 b	14.51 ± 0.00 a
6	15.65 ± 0.00 d	4.28 ± 0.02 d	63.30 ± 0.02 b	13.83 ± 0.00 b
12	15.71 ± 0.03 d	4.28 ± 0.02 d	63.33 ± 0.03 b	13.89 ± 0.02 b
18	15.97 ± 0.00 bc	4.59 ± 0.01 ab	62.32 ± 0.06 d	13.33 ± 0.02 c
24	15.95 ± 0.01 c	4.51 ± 0.01 abc	62.49 ± 0.01 c	13.31 ± 0.01 c
30	15.99 ± 0.02 abc	4.62 ± 0.04 a	61.60 ± 0.00 d	12.60 ± 0.00 e
36	15.92 ± 0.04 c	4.34 ± 0.05 cd	64.01 ± 0.05 a	12.70 ± 0.01 d
42	16.06 ± 0.00 a	4.27 ± 0.06 d	64.02 ± 0.05 a	11.34 ± 0.00 h
48	16.08 ± 0.04 a	4.34 ± 0.13 cd	64.04 ± 0.00 a	11.31 ± 0.01 h
54	16.08 ± 0.05 a	4.41 ± 0.10 bcd	63.31 ± 0.03 b	11.83 ± 0.02 g
60	16.06 ± 0.02 ab	4.29 ± 0.02 d	63.46 ± 0.06 f	12.00 ± 0.04 f

Results are means ± SD of fatty acid composition. Row values in the same column bearing a different letter are significantly different (*p* < 0.05).

**Table 2:** The differences in LF-NMR relaxation properties of GLSO during a 60 d storage period.

Time (d)	T_21_ (ms)	S21 (%)	T_22_ (ms)	S22 (%)
0	83.10	95.60	439.76	4.40
6	83.10	95.64	471.38	4.36
12	77.53	94.94	410.27	5.06
18	77.53	94.94	410.27	5.06
24	72.33	95.22	382.75	4.78
30	77.53	95.20	439.76	4.80
36	72.33	95.23	382.75	4.77
42	62.95	96.59	357.08	3.41
48	58.73	96.44	333.13	3.56
54	67.48	94.84	382.75	5.16
60	72.33	95.07	410.27	4.94

**Table 3:** Assignment to different kinds of protons of some of the major signals, shown in Figure 4, appeared in the ^1^H NMR spectra of GLSO.

Signal	Chemical Shift (ppm)	Functional Group
I	4.10–4.32 (dd,dd)	–C**H**_2_OCOR (glyceryl groups)
**Conjugated dienic systems**		
A	6.27 (ddm)	–C**H**=C**H**–C**H**=C**H**– ((E,E)-conjugated dienes)
B	6.49 (dd)	–C**H**=C**H**–C**H**=C**H**– ((Z,E)-conjugated dienes)
**Epoxides**		
C	2.63 (m)	–C**H**O**H**C– ((E)-9,10-epoxystearyl groups)
D	2.88 (m)	–C**H**O**H**C– ((Z)-9,10-epoxystearyl groups)
**Aldehydes**		
E	9.49 (d)	–C**H**O ((E)-2-alkenals)
F	9.52 (d)	–C**H**O ((E,E)-2,4-alkadienals)
G	9.58 (d)	–C**H**O (4-hydroxy-(E)-2-alkenals)
H	9.75 (t)	–C**H**O (n-alkanals)

Abbreviations: m, multiplet; d, doublet; t, triplet; dd,dd, double doublet.

**Table 4:** Changes in relative concentrations of volatile compounds in GLSO during 60 d storage period.

Compounds	RI	Concentration										
		0 d	6 d	12	18	24	30	36	42	48	54	60
**Aldehyde**												
Pentanal	670	1.36 ± 0.14 d	2.05 ± 0.26 abcd	2.4 ± 0.3 ab	1.84 ± 0.25 bcd	1.47 ± 0.22 cd	2.15 ± 0.18 abc	1.53 ± 0.34 cd	2.04 ± 0.12 abcd	2.74 ± 0.06 a	2.11 ± 0.17 abc	2.48 ± 0.05 ab
2-methyl-Pentanal	938	0.06 ± 0.01 c	0.27 ± 0.07 a	0.24 ± 0.01 ab	0.15 ± 0.03 bc	0.11 ± 0.01 c	0.16 ± 0.05 abc	0.1 ± 0.04 c	0.11 ± 0.02 c	0.13 ± 0.04 bc	0.08 ± 0.02 c	0.08 ± 0.01 c
Hexanal	771	7.31 ± 0.63 f	16.46 ± 0.74 bc	19.26 ± 0.82 a	15.8 ± 1.16 bc	12.43 ± 0.46 de	19.76 ± 0.92 a	11.8 ± 0.29 e	15.66 ± 0.53 bc	16.73 ± 0.76 b	13 ± 0.34 de	14.38 ± 0.3 cd
2-Hexenal	850	—	0.04 ± 0.01 bc	0.05 ± 0.01 abc	0.03 ± 0.01 cd	0.03 ± 0 cd	0.04 ± 0.01 bc	0.03 ± 0 cd	0.05 ± 0 abc	0.08 ± 0.03 a	0.07 ± 0 ab	0.07 ± 0.01 ab
Heptanal	879	—	0.12 ± 0.05 ab	0.12 ± 0.03 ab	0.13 ± 0.04 ab	0.11 ± 0.03 ab	0.12 ± 0.02 ab	0.16 ± 0.05 ab	0.17 ± 0.06 a	0.16 ± 0.03 ab	0.19 ± 0.03 a	0.27 ± 0.1 a
(E)-2-Heptenal	950	0.12 ± 0.03 c	0.55 ± 0.13 bc	0.71 ± 0.22 ab	0.55 ± 0.17 bc	0.52 ± 0.12 bc	0.71 ± 0.24 ab	0.56 ± 0.18 bc	0.59 ± 0.1 abc	0.7 ± 0.05 ab	0.76 ± 0.17 ab	1.1 ± 0.08 a
Octanal	983	0.18 ± 0 b	0.16 ± 0 b	0.33 ± 0.02 bc	0.31 ± 0 bc	0.28 ± 0.02 b	0.34 ± 0.03 bc	0.41 ± 0.02 bc	0.51 ± 0.03 bc	0.52 ± 0.12 bc	0.53 ± 0.02 bc	0.71 ± 0.02 a
(E)-2-Octenal	1039	0.02 ± 0 f	0.17 ± 0.04 cde	0.32 ± 0 b	0.11 ± 0.04 def	0.28 ± 0.03 bc	0.13 ± 0.04 def	0.24 ± 0.08 bcd	0.48 ± 0.06 a	0.17 ± 0.05 cde	0.03 ± 0 f	0.06 ± 0 ef
(E)-4-Nonenal	1180	—	0.02 ± 0 c	0.03 ± 0.01 c	0.02 ± 0 c	0.02 ± 0 c	0.11 ± 0.03 a	0.09 ± 0.03 ab	0.09 ± 0.02 ab	0.13 ± 0.03 a	0.04 ± 0.01 bc	0.03 ± 0 c
Nonanal	1087	0.38 ± 0.07 b	0.55 ± 0.1 b	0.55 ± 0.07 b	0.54 ± 0.11 b	0.45 ± 0.07 b	0.75 ± 0.14 ab	0.64 ± 0.2 b	0.88 ± 0.27 ab	0.73 ± 0.22 ab	0.84 ± 0.15 ab	1.24 ± 0.27 a
(E)-2-Nonenal	1127	—	0.02 ± 0 bcd	0.02 ± 0 bcd	0.02 ± 0 bcd	0.01 ± 0 cd	0.04 ± 0.02 ab	0.03 ± 0 bc	0.04 ± 0 ab	0.03 ± 0 bc	0.04 ± 0.01 ab	0.06 ± 0.01 a
Decanal	1189	0.02 ± 0.01 c	0.02 ± 0 c	0.03 ± 0 bc	0.03 ± 0 bc	0.03 ± 0 bc	0.08 ± 0.02 ab	0.06 ± 0.02 abc	0.08 ± 0.02 ab	0.1 ± 0.03 a	0.07 ± 0 abc	0.11 ± 0.03 a
(E,Z)-2,4-Decadienal	1460	—	—	0.07 ± 0.02 bcd	0.1 ± 0.01 bc	0.12 ± 0.03 b	0.24 ± 0.07 a	0.03 ± 0 cd	0.05 ± 0.02 bcd	0.1 ± 0.01 bc	0.06 ± 0.02 bcd	0.08 ± 0 bcd
**Ketone**												
2-Heptanone	868	0.15 ± 0.02 bc	0.14 ± 0.07 c	0.18 ± 0.05 bc	0.24 ± 0.02 bc	0.21 ± 0.04 bc	0.34 ± 0.13 abc	0.32 ± 0.01 bc	0.36 ± 0.04 abc	0.31 ± 0.04 bc	0.37 ± 0.04 ab	0.54 ± 0.12 a
1-Octen-3-one	980	—	—	—	—	—	0.04 ± 0.02 c	0.03 ± 0 c	0.67 ± 0.08 b	1.14 ± 0.08 a	0.81 ± 0.2 b	0.04 ± 0 c
3-Octen-2-one	990	0.04 ± 0 bc	0.03 ± 0 c	0.04 ± 0 bc	0.05 ± 0 bc	0.03 ± 0 c	0.03 ± 0.01 c	0.04 ± 0.01 bc	0.07 ± 0.02 abc	0.1 ± 0.02 a	0.08 ± 0.01 ab	0.1 ± 0.02 a
trans-3-Nonen-2-one	1100	—	—	—	—	—	0.11 ± 0.02 a	0.08 ± 0.02 a	0.08 ± 0.02 a	0.09 ± 0.02 a	0.1 ± 0.02 a	0.11 ± 0.03 a
**Alcohol**												
1-Pentanol	746	0.14 ± 0.03 c	0.39 ± 0.05 abc	0.46 ± 0.06 abc	0.39 ± 0.08 abc	0.32 ± 0.07 bc	0.54 ± 0.16 ab	0.41 ± 0.09 abc	0.41 ± 0.11 abc	0.76 ± 0.21 a	0.5 ± 0.07 abc	0.69 ± 0.16 ab
1-Heptanol	958	—	—	—	—	—	0.11 ± 0.01 ab	0.08 ± 0.04 bc	0.07 ± 0.02 bc	0.05 ± 0 cd	0.1 ± 0.02 bc	0.16 ± 0.02 a
1-Octen-3-ol	980	0.22 ± 0.03 b	0.61 ± 0.15 bc	0.75 ± 0.16 bc	0.61 ± 0.12 bc	0.46 ± 0.05 bc	0.74 ± 0.35 bc	0.57 ± 0.12 bc	0.49 ± 0.04 bc	0.56 ± 0.16 bc	0.61 ± 0.08 bc	0.97 ± 0.13 a
1-Octanol	1060	—	0.06 ± 0.02 ab	0.09 ± 0.03 a	0.07 ± 0.02 a	0.06 ± 0.02 ab	0.09 ± 0.03 a	0.07 ± 0.01 a	0.06 ± 0.02 ab	0.1 ± 0.02 a	0.07 ± 0.02 a	0.12 ± 0.01 a
**Alkanes**												
Heptane	700	—	0.07 ± 0.02 cd	0.07 ± 0.02 cd	0.11 ± 0.03 bc	0.09 ± 0.02 cd	0.1 ± 0 bc	0.08 ± 0.01 cd	0.14 ± 0.05 abc	0.23 ± 0.02 a	0.19 ± 0.05 ab	0.13 ± 0.03 bc
Octane	948	—	0.05 ± 0 de	0.1 ± 0.02 de	0.07 ± 0 de	0.13 ± 0.02 cde	0.11 ± 0.03 de	0.13 ± 0.02 cde	0.26 ± 0.03 bc	0.41 ± 0.12 a	0.3 ± 0.02 ab	0.17 ± 0.02 bcd
**carboxylic acid**												
Hexanoic acid	972	2.29 ± 0.2 bcde	1.91 ± 0.37 cde	1.85 ± 0.24 de	1.78 ± 0.23 de	1.45 ± 0 e	1.96 ± 0.23 cde	2.56 ± 0.27 bcd	2.53 ± 0.32 bcd	2.82 ± 0.35 bc	2.9 ± 0.28 b	4.05 ± 0.25 a
Octanoic acid	1180	0.12 ± 0.01 ab	0.11 ± 0.02 ab	0.13 ± 0.03 ab	0.13 ± 0.04 ab	0.11 ± 0.02 ab	0.1 ± 0.04 ab	0.06 ± 0.01 b	0.08 ± 0.02 b	0.09 ± 0.02 ab	0.11 ± 0.02 ab	0.17 ± 0.02 a
Nonanoic acid	1253	0.25 ± 0.04 cde	0.6 ± 0.09 a	0.51 ± 0.05 abc	0.33 ± 0.05 bcde	0.43 ± 0.13 abc	0.13 ± 0.04 de	0.1 ± 0.02 e	0.3 ± 0.05 bcde	0.56 ± 0.15 ab	0.38 ± 0.01 abcd	0.42 ± 0.09 abc
**Furanone**												
dihydro-5-pentyl-2(3H)-Furanone	1300	—	—	—	—	—	—	0.03 ± 0.01 bc	0.04 ± 0.01 b	0.08 ± 0.02 a	0.05 ± 0 ab	0.06 ± 0.02 ab

Note: “—“, non-detectable. RI, retention index. Different lowercase letters indicated that there were significant differences among the samples prepared with the different times (*p* < 0.05).

## Data Availability

The original contributions presented in the study are included in the article; further inquiries can be directed to the corresponding author.
